# Comparison of Intercom and Megaphone Hashtags Using Four Years of Tweets From the Top 44 Schools of Nursing: Thematic Analysis

**DOI:** 10.2196/25114

**Published:** 2021-04-20

**Authors:** Kimberly Acquaviva

**Affiliations:** 1 School of Nursing University of Virginia Charlottesville, VA United States

**Keywords:** Twitter, hashtag, nurses, media, intercom hashtag, megaphone hashtag

## Abstract

**Background:**

When this study began in 2018, I sought to determine the extent to which the top 50 schools of nursing were using hashtags that could attract attention from journalists on Twitter. In December 2020, the timeframe was expanded to encompass 2 more years of data, and an analysis was conducted of the types of hashtags used.

**Objective:**

The study attempted to answer the following question: to what extent are top-ranked schools of nursing using hashtags that could attract attention from journalists, policy makers, and the public on Twitter?

**Methods:**

In February 2018, 47 of the top 50 schools of nursing had public Twitter accounts. The most recent 3200 tweets were extracted from each account and analyzed. There were 31,762 tweets in the time period covered (September 29, 2016, through February 22, 2018). After 13,429 retweets were excluded, 18,333 tweets remained. In December 2020, 44 of the original 47 schools of nursing still had public Twitter accounts under the same name used in the first phase of the study. Three accounts that were no longer active were removed from the 2016-2018 data set, resulting in 16,939 tweets from 44 schools of nursing. The Twitter data for the 44 schools of nursing were obtained for the time period covered in the second phase of the study (February 23, 2018, through December 13, 2020), and the most recent 3200 tweets were extracted from each of the accounts. On excluding retweets, there were 40,368 tweets in the 2018-2020 data set. The 2016-2018 data set containing 16,939 tweets was merged with the 2018-2020 data set containing 40,368 tweets, resulting in 57,307 tweets in the 2016-2020 data set.

**Results:**

Each hashtag used 100 times or more in the 2016-2020 data set was categorized as one of the following seven types: nursing, school, conference or tweet chat, health, illness/disease/condition, population, and something else. These types were then broken down into the following two categories: intercom hashtags and megaphone hashtags. Approximately 83% of the time, schools of nursing used intercom hashtags (inward-facing hashtags focused on in-group discussion within and about the profession). Schools of nursing rarely used outward-facing megaphone hashtags. There was no discernible shift in the way that schools of nursing used hashtags after the publication of *The Woodhull Study Revisited*.

**Conclusions:**

Top schools of nursing use hashtags more like intercoms to communicate with other nurses rather than megaphones to invite attention from journalists, policy makers, and the public. If schools of nursing want the media to showcase their faculty members as experts, they need to increase their use of megaphone hashtags to connect the work of their faculty with topics of interest to the public.

## Introduction

Twitter is a microblogging website where users can post “tweets” (brief messages, images, and videos) to share with “followers” (people who have chosen to follow their Twitter account). Hashtags are words or phrases (without spaces) that are preceded by a pound sign (#) [1]. Hashtags first came into use on Twitter in 2007 when a user named Chris Messina put forward a proposal for “…improving contextualization, content filtering, and exploratory serendipity within Twitter” [2]. In his proposal, Messina wrote that his primary interest was “simply having a better eavesdropping experience on Twitter” [2]. In 2018, hashtags were widely used on Twitter to make tweets easy to find for other Twitter users interested in a given topic.

When the landmark *Woodhull Study on Nursing and the Media* was published in 1998, the voices and faces of nurses were found to be largely absent from news stories [3]. Mary Chaffee wrote that “[t]his lack of visibility limits nursing’s ability to communicate important health information, impedes nursing’s ability to define its role and contributions in the health care delivery system, and restricts nursing’s ability to advocate for health policy” [4]. Because Twitter was not launched until 8 years after the Woodhull Study was conducted, the researchers obviously could not look at Twitter data in their analysis. Shattell and Darmoc argue that nurses should consider using Twitter to make their “practical, real-life knowledge or…research findings or insights on current issues… available for the public” and to “harness attention from some more traditional media sources” [5]. While there is an abundance of research regarding the use of hashtags by health care professionals on Twitter [6-10], little is known about the ways in which schools of nursing used Twitter to invite attention from and engagement with journalists, policy makers, and the general public in the 2 years before *The Woodhull Study Revisited* was published in September 2018 and the 2 years after its publication. This study seeks to fill this gap.

When this study began in 2018 as a last-minute addition to *The Woodhull Study Revisited*, I sought to determine the extent to which the top 50 schools of nursing were using hashtags that could attract/invite attention from journalists on Twitter [11]. Preliminary findings using 2016-2018 data were intriguing but were not published with the rest of the results of *The Woodhull Study Revisited* [12]. In December 2020, the timeframe was expanded to encompass 2 more years of data so that before and after *Woodhull Study Revisited* analyses could be conducted. In addition, the scope was expanded to include an in-depth analysis of the types of hashtags used by schools of nursing. The resulting study is a comprehensive analysis of 4 years of tweets from the top 44 schools of nursing in the United States.

Methods have been described in detail using plain language so that researchers can easily replicate the study without needing specialized knowledge in natural language processing or machine learning. Democratizing Twitter analysis requires greater transparency regarding the methods used. As such, each table in this manuscript illustrates a step in the data analysis process that would otherwise be opaque to readers if the step was simply described in the narrative.

## Methods

### Research Question

The study sought to answer the following question: to what extent are top-ranked schools of nursing using hashtags that could attract/invite attention from journalists, policy makers, and the general public on Twitter? Below is a detailed description of the methods used for sampling, data collection, and data analysis.

### Sampling

When this study began in February 2018, the sample of nursing schools was drawn from US News and World Report’s 2017 list of the top nursing schools with master’s degree programs. Fifty of the highest-ranked schools were selected from this list, with numerical rankings ranging from 1st to 48th (with several ties). The US News and World Report rankings were used as a mechanism for identifying the schools of nursing to include in this study with the knowledge that the rankings do not necessarily mean that the schools included at the top of the list are inherently “better” than the schools ranked lower. The decision to include the 50 highest-ranked schools of nursing in the sample was based on the fact that the US News and World Report rankings are the primary way that members of the media can quickly identify top schools of nursing nationally. The US News and World Report gets 7 million unique visitors to the education rankings and information webpages each month (US News and World Report, 2018).

In February 2018, of US News and World Report’s 50 top schools of nursing, two schools did not have a Twitter account and one school had a locked private Twitter account that was inaccessible to anyone other than those who were given permission by the school to follow the account. Thus, the school of nursing with the locked Twitter account and the two schools without a Twitter account were excluded from the 2016-2018 data set. The three schools omitted from the 2016-2018 data set are indicated in Table 1. In December 2020, when the second phase of this study was conducted, 44 of the original 47 schools of nursing still had public Twitter accounts under the same name used in the 2016-2018 data set. The three schools that no longer had a public Twitter account under the same name in 2020 are indicated in Table 1 and were omitted from both the 2016-2018 and 2018-2020 data sets for the sake of consistency.

**Table 1:** Sample composition.

2017 US News & World Report Rank	Name of the university	Name of the school of nursing	Official school Twitter account in February 2018	Account status in December 2020
#1	Duke University	School of Nursing	@DukeU_NrsngSchl	Active
#2	Johns Hopkins University	School of Nursing	@JHUNursing	Active
#3	University of Pennsylvania	Penn Nursing Science	@PennNursing	Active
#4	Emory University	Nell Hodgson Woodruff School of Nursing	@EmoryNursing	Active
#5	Ohio State University	College of Nursing	@osunursing	Active
#6 Tie	University of Washington	School of Nursing	@UWSoN	Active
#6 Tie	Yale University	School of Nursing	@YaleNursing	Active
#8 Tie^a^	Columbia University	School of Nursing	@CU_Nursing	Inactive
#8 Tie	University of Pittsburgh	School of Nursing	@UPittNursing	Active
#10	University of Maryland–Baltimore	School of Nursing	@MarylandNursing	Active
#11 Tie	Case Western Reserve University	Frances Payne Bolton School of Nursing	@fpbnursing	Active
#11 Tie	University of Michigan–Ann Arbor	School of Nursing	@UMichNursing	Active
#13 Tie	New York University (Meyers)	Rory Myers College of Nursing	@NYUNursing	Active
#13 Tie	University of Alabama–Birmingham	School of Nursing	@UABSON	Active
#15 Tie	University of California Los Angeles	School of Nursing	@UCLANursing	Active
#15 Tie	Vanderbilt University	School of Nursing	@VanderbiltNurse	Active
#17	University of North Carolina–Chapel Hill	School of Nursing	@UNCSON	Active
#18	Rush University	College of Nursing	@RushUNursing	Active
#19	University of Virginia	School of Nursing	@UVASON	Active
#20 Tie	Pennsylvania State University–University Park	College of Nursing	@PSUNursing	Active
#20 Tie	Rutgers University–Newark	School of Nursing	@RU_Nursing	Active
#20 Tie	University of Illinois–Chicago	College of Nursing	@UICnursing	Active
#23 Tie^a^	University of Iowa	College of Nursing	@UICollegeofNurs	Inactive
#23 Tie	University of Texas–Austin	School of Nursing	@LonghornNursing	Active
#23 Tie^b^	University of Texas Health Science Center–Houston	Cizik School of Nursing	No Twitter account found	N/A^c^
#26 Tie^b^	Medical University of South Carolina	College of Nursing	@MUSC_CON Locked account	N/A
#26 Tie	University of Colorado Anschutz Medical Campus	College of Nursing	@NursingCU	Active
#28 Tie	Georgetown University	School of Nursing and Health Studies	@GtownNHS	Active
#28 Tie	Indiana University-Purdue University–Indianapolis	School of Nursing	@IUSONIndy	Active
#28 Tie^b^	University of San Diego	Hahn School of Nursing and Health Science	No Twitter account found	N/A
#31 Tie	Arizona State University	College of Nursing and Health Innovation	@asunursing	Active
#31 Tie	Boston College	Connell School of Nursing	@BC_CSON	Active
#31 Tie	The Catholic University of America	School of Nursing	@CUANursing	Active
#31 Tie	George Washington University	School of Nursing	@GWNursing	Active
#31 Tie	University of Utah	College of Nursing	@uofunursing	Active
#36 Tie	Oregon Health and Science University	School of Nursing	@OHSUNursing	Active
#36 Tie	University of Rochester	School of Nursing	@UofRSON	Active
#38 Tie	University of Cincinnati	College of Nursing	@UCnursing	Active
#38 Tie	University of Miami	School of Nursing and Health Studies	@UMiamiNursing	Active
#38 Tie	University of Missouri	Sinclair School of Nursing	@MizzouNursing	Active
#41 Tie^a^	University of Arizona	College of Nursing	@UACON	Inactive
#41 Tie	Washington State University	College of Nursing	@WSUNursing	Active
#43 Tie	University of Connecticut	School of Nursing	@UConnNursing	Active
#43 Tie	University of Missouri–Kansas City	School of Nursing and Health Studies	@UMKCSoNHS	Active
#45 Tie	Florida Atlantic University (Lynn)	Christine E. Lynn College of Nursing	@faunursing	Active
#45 Tie	University of Massachusetts–Amherst	College of Nursing	@UMAnursing	Active
#48 Tie	University of Alabama	Capstone College of Nursing	@uaccn	Active
#48 Tie	University of Tennessee–Knoxville	College of Nursing	@utknursing	Active
#48 Tie	Virginia Commonwealth University	School of Nursing	@VCUNursing	Active
#48 Tie	Wayne State University	College of Nursing	@WSUCoN	Active

^a^Schools that no longer had a public Twitter account under the same name in 2020.

^b^Schools omitted from the 2016-2018 data set.

^c^N/A: not applicable.

### Data Collection

Data collection was conducted twice during this study. In February 2018, a list of the top 50 schools of nursing was matched with publicly accessible Twitter accounts and then a data request was submitted to Export Tweet for the most recent 3200 tweets from each of the top-ranked schools of nursing. Because schools of nursing tweet with varying frequency, the past 3200 tweets for any given school of nursing covered a wide array of time frames. At one end of the spectrum, there were five schools of nursing, including Vanderbilt University, Johns Hopkins University, University of Michigan–Ann Arbor, Boston College, and University of Pennsylvania, for whom the oldest tweet in the data set was from 2016. At the other end of the spectrum, there were five schools of nursing, including University of Virginia, Yale University, Case Western Reserve University, University of Utah, and University of North Carolina–Chapel Hill, for whom the oldest tweet was from early 2009. Table 2 lists the oldest tweet in the data set from each school, with schools of nursing listed in order of their oldest tweet in the data set.

**Table 2:** Oldest tweets in the 2016-2018 data set.

Name of the university	Official school Twitter account	Date of the oldest tweet in the 2016-2018 data set
University of Virginia	@UVASON	March 02, 2009
Yale University	@YaleNursing	March 10, 2009
Case Western Reserve University	@fpbnursing	March 12, 2009
University of Utah	@uofunursing	May 5, 2009
University of North Carolina–Chapel Hill	@UNCSON	May 7, 2009
University of California Los Angeles	@UCLANursing	August 7, 2009
New York University (Meyers)	@NYUNursing	October 27, 2009
University of Missouri–Kansas City	@UMKCSoNHS	December 07, 2009
University of Illinois–Chicago	@UICnursing	January 4, 2010
Arizona State University	@asunursing	January 19, 2010
Washington State University	@WSUNursing	January 29, 2010
Florida Atlantic University (Lynn)	@faunursing	April 22, 2010
University of Miami	@UMiamiNursing	April 30, 2010
George Washington University	@GWNursing	September 29, 2010
University of Alabama–Birmingham	@UABSON	May 12, 2011
Wayne State University	@WSUCoN	June 21, 2011
Indiana University-Purdue University–Indianapolis	@IUSONIndy	July 13, 2011
University of Washington	@UWSoN	July 26, 2011
Emory University	@EmoryNursing	February 10, 2012
Oregon Health and Science University	@OHSUNursing	February 18, 2012
Georgetown University	@GtownNHS	March 12, 2012
Ohio State University	@osunursing	April 12, 2012
University of Alabama	@uaccn	April 24, 2012
Duke University	@DukeU_NrsngSchl	May 11, 2012
University of Massachusetts–Amherst	@UMAnursing	June 12, 2012
University of Tennessee–Knoxville	@utknursing	July 17, 2012
Rush University	@RushUNursing	July 27, 2012
University of Maryland–Baltimore	@MarylandNursing	August 10, 2012
University of Missouri	@MizzouNursing	May 13, 2013
University of Rochester	@UofRSON	October 28, 2013
University of Colorado Anschutz Medical Campus	@NursingCU	February 28, 2014
University of Pittsburgh	@UPittNursing	March 18, 2014
Rutgers University–Newark	@RU_Nursing	April 30, 2014
University of Cincinnati	@UCnursing	June 17, 2014
Pennsylvania State University–University Park	@PSUNursing	October 23, 2014
University of Connecticut	@UConnNursing	November 30, 2014
Virginia Commonwealth University	@VCUNursing	January 27, 2015
University of Texas–Austin	@LonghornNursing	April 9, 2015
The Catholic University of America	@CUANursing	April 10, 2015
University of Pennsylvania	@PennNursing	March 24, 2016
Boston College	@BC_CSON	April 7, 2016
University of Michigan–Ann Arbor	@UMichNursing	June 16, 2016
Johns Hopkins University	@JHUNursing	July 22, 2016
Vanderbilt University	@VanderbiltNurse	September 29, 2016

Table 2 was used to determine the most recent “oldest tweet” date in the 2016-2018 data set. The @VanderbiltNurse Twitter account had the most recent “oldest tweet” (September 29, 2016), so September 29, 2016, was selected as the start date for the analysis. This meant that the time period to be covered in the 2016-2018 data set would be September 29, 2016, through February 22, 2018. Tweets with dates older than September 29, 2016, were filtered out from the data set, resulting in 16,939 tweets for the 2016-2018 data set. Table 3 describes the composition of the final 2016-2018 data set, with schools listed in alphabetical order by Twitter account name.

**Table 3:** Composition of the 2016-2018 data set.

Name of the university	Official school Twitter account	Number of tweets
Arizona State University	@asunursing	430
Boston College	@BC_CSON	138
The Catholic University of America	@CUANursing	7
Duke University	@DukeU_NrsngSchl	415
Emory University	@EmoryNursing	437
Florida Atlantic University (Lynn)	@faunursing	303
Case Western Reserve University	@fpbnursing	159
Georgetown University	@GtownNHS	257
George Washington University	@GWNursing	883
Indiana University-Purdue University–Indianapolis	@IUSONIndy	251
Johns Hopkins University	@JHUNursing	1992
University of Texas–Austin	@LonghornNursing	545
University of Maryland–Baltimore	@MarylandNursing	738
University of Missouri	@MizzouNursing	49
University of Colorado Anschutz Medical Campus	@NursingCU	206
New York University (Meyers)	@NYUNursing	184
Oregon Health and Science University	@OHSUNursing	312
Ohio State University	@osunursing	949
University of Pennsylvania	@PennNursing	1342
Pennsylvania State University–University Park	@PSUNursing	94
Rutgers University–Newark	@RU_Nursing	88
Rush University	@RushUNursing	191
University of Alabama–Birmingham	@UABSON	390
University of Alabama	@uaccn	166
University of California–Los Angeles	@UCLANursing	99
University of Cincinnati	@UCnursing	318
University of Connecticut	@UConnNursing	20
University of Illinois–Chicago	@UICnursing	124
University of Massachusetts–Amherst	@UMAnursing	38
University of Miami	@UMiamiNursing	39
University of Michigan–Ann Arbor	@UMichNursing	942
University of Missouri–Kansas City	@UMKCSoNHS	31
University of North Carolina–Chapel Hill	@UNCSON	80
University of Rochester	@UofRSON	587
University of Utah	@uofunursing	138
University of Pittsburgh	@UPittNursing	179
University of Tennessee–Knoxville	@utknursing	208
University of Virginia	@UVASON	120
University of Washington	@UWSoN	152
Vanderbilt University	@VanderbiltNurse	2692
Virginia Commonwealth University	@VCUNursing	107
Wayne State University	@WSUCoN	42
Washington State University	@WSUNursing	265
Yale University	@YaleNursing	232

During phase two of the study, a data request was submitted to Vicinitas for all tweets from February 23, 2018, through December 13, 2020, from the 44 still-active Twitter accounts. Tweets prior to February 23, 2018, were deleted from the data set. Table 4 lists the oldest tweet in the 2018-2020 data set from each school, along with the number of tweets per school.

**Table 4:** Oldest tweet and total tweets from each school in the 2018-2020 data set.

Name of the university	Official school Twitter account	Oldest tweet date	Total number of tweets
University of Virginia	@UVASON	February 28, 2018	914
Yale University	@YaleNursing	February 23, 2018	550
Case Western Reserve University	@fpbnursing	February 23, 2018	701
University of Utah	@uofunursing	February 23, 2018	707
University of North Carolina–Chapel Hill	@UNCSON	February 23, 2018	396
University of California–Los Angeles	@UCLANursing	February 28, 2018	446
New York University (Meyers)	@NYUNursing	February 23, 2018	655
University of Missouri–Kansas City	@UMKCSoNHS	March 1, 2018	105
University of Illinois–Chicago	@UICnursing	February 27, 2018	523
Arizona State University	@asunursing	February 23, 2018	1943
Washington State University	@WSUNursing	February 23, 2018	504
Florida Atlantic University (Lynn)	@faunursing	February 23, 2018	565
University of Miami	@UMiamiNursing	February 27, 2018	445
George Washington University	@GWNursing	February 23, 2018	2056
University of Alabama–Birmingham	@UABSON	February 28, 2018	990
Wayne State University	@WSUCoN	February 27, 2018	141
Indiana University-Purdue University–Indianapolis	@IUSONIndy	February 25, 2018	445
University of Washington	@UWSoN	February 23, 2018	822
Emory University	@EmoryNursing	February 23, 2018	859
Oregon Health and Science University	@OHSUNursing	February 23, 2018	375
Georgetown University	@GtownNHS	February 23, 2018	961
Ohio State University	@osunursing	February 23, 2018	1927
University of Alabama	@uaccn	March 1, 2018	210
Duke University	@DukeU_NrsngSchl	February 23, 2018	900
University of Massachusetts–Amherst	@UMAnursing	April 27, 2018	53
University of Tennessee–Knoxville	@utknursing	February 23, 2018	577
Rush University	@RushUNursing	March 1, 2018	334
University of Maryland–Baltimore	@MarylandNursing	February 26, 2018	1348
University of Missouri	@MizzouNursing	February 26, 2018	258
University of Rochester	@UofRSON	February 23, 2018	558
University of Colorado Anschutz Medical Campus	@NursingCU	February 23, 2018	595
University of Pittsburgh	@UPittNursing	February 23, 2018	400
Rutgers University–Newark	@RU_Nursing	February 27, 2018	462
University of Cincinnati	@UCnursing	February 24, 2018	509
Pennsylvania State University–University Park	@PSUNursing	February 23, 2018	600
University of Connecticut	@UConnNursing	February 27, 2018	136
Virginia Commonwealth University	@VCUNursing	February 26, 2018	240
University of Texas–Austin	@LonghornNursing	February 25, 2018	795
The Catholic University of America	@CUANursing	March 9, 2018	1
University of Pennsylvania	@PennNursing	February 23, 2018	2357
Boston College	@BC_CSON	February 23, 2018	281
University of Michigan–Ann Arbor	@UMichNursing	February 23, 2018	1435
Johns Hopkins University	@JHUNursing	February 23, 2018	6570
Vanderbilt University	@VanderbiltNurse	February 23, 2018	4719

After cleaning the data, the 2016-2018 and 2018-2020 data sets were merged into a single data set containing 57,307 tweets. Table 5 describes the composition of the new 2016-2020 data set, with schools listed in alphabetical order by Twitter account name.

In December 2020, the original list of 47 schools of nursing was matched with publicly accessible Twitter accounts. Of the original 47 schools of nursing, 44 still had public Twitter accounts under the same name used in the first part of the study. The three Twitter accounts that were no longer active (@UICollegeofNurs, @UACON, and @CU_Nursing) were removed from the original data set, resulting in a data set containing 16,939 tweets from 44 top-ranked schools of nursing. The most recent 3200 tweets from each of the Twitter accounts were extracted and analyzed. Excluding retweets, there were 40,368 tweets for the time period covered (February 23, 2018, through December 13, 2020). These 40,368 tweets were added to the data set, resulting in a data set containing 57,307 tweets from September 29, 2016, through December 13, 2020.

**Table 5:** Composition of the final 2016-2020 data set.

Name of the university	Official school Twitter account	Number of tweets in the 2016-2018 data set	Number of tweets in the 2018-2020 data set	Total number of tweets in the 2016-2020 data set
Arizona State University	@asunursing	430	1943	2373
Boston College	@BC_CSON	138	281	419
The Catholic University of America	@CUANursing	7	701	708
Duke University	@DukeU_NrsngSchl	415	900	1315
Emory University	@EmoryNursing	437	859	1296
Florida Atlantic University (Lynn)	@faunursing	303	565	868
Case Western Reserve University	@fpbnursing	159	2056	2215
Georgetown University	@GtownNHS	257	961	1218
George Washington University	@GWNursing	883	445	1328
Indiana University-Purdue University–Indianapolis	@IUSONIndy	251	6570	6821
Johns Hopkins University	@JHUNursing	1992	655	2647
University of Texas–Austin	@LonghornNursing	545	1927	2472
University of Maryland–Baltimore	@MarylandNursing	738	375	1113
University of Missouri	@MizzouNursing	49	600	649
University of Colorado Anschutz Medical Campus	@NursingCU	206	334	540
New York University (Meyers)	@NYUNursing	184	462	646
Oregon Health and Science University	@OHSUNursing	312	1	313
Ohio State University	@osunursing	949	210	1159
University of Pennsylvania	@PennNursing	1342	990	2332
Pennsylvania State University–University Park	@PSUNursing	94	446	540
Rutgers University–Newark	@RU_Nursing	88	509	597
Rush University	@RushUNursing	191	595	786
University of Alabama–Birmingham	@UABSON	390	136	526
University of Alabama	@uaccn	166	523	689
University of California–Los Angeles	@UCLANursing	99	1348	1447
University of Cincinnati	@UCnursing	318	53	371
University of Connecticut	@UConnNursing	20	445	465
University of Illinois–Chicago	@UICnursing	124	1435	1559
University of Massachusetts–Amherst	@UMAnursing	38	258	296
University of Miami	@UMiamiNursing	39	105	144
University of Michigan–Ann Arbor	@UMichNursing	942	396	1338
University of Missouri–Kansas City	@UMKCSoNHS	31	2357	2388
University of North Carolina–Chapel Hill	@UNCSON	80	400	480
University of Rochester	@UofRSON	587	558	1145
University of Utah	@uofunursing	138	577	715
University of Pittsburgh	@UPittNursing	179	795	974
University of Tennessee–Knoxville	@utknursing	208	707	915
University of Virginia	@UVASON	120	914	1034
University of Washington	@UWSoN	152	822	974
Vanderbilt University	@VanderbiltNurse	2692	4719	7411
Virginia Commonwealth University	@VCUNursing	107	240	347
Wayne State University	@WSUCoN	42	504	546
Washington State University	@WSUNursing	265	141	406
Yale University	@YaleNursing	232	550	782

### Data Analysis

The analyses in this study were conducted using R version 4.0.3 (Bunny-Wunnies Freak Out), R Studio Version 1.3.1093, and Microsoft Excel for Mac Version 16.43. The following are the steps taken to generate a list of the most frequently used hashtags in the 2016-2020 data set, along with the number of times each hashtag appeared. Initially, the Excel file was uploaded to R software. The R Markdown package was installed, and the elements of Van Horn and Beveridge coding were used [13]. The text strings in the data set were cleaned. The character encoding in tweets was homogenized to remove the strings of nonsense characters indicating the presence of emojis in the source tweets. This converted character encoding to Unicode UTF-8. Thereafter, capitalization in tweets was removed by turning everything into lowercase. Subsequently, extra whitespace and URLs were removed from the tweets. Once the text strings were cleaned, the hashtags present in the data set were identified and a list of the hashtags from most to least frequently used was generated. The data frame generated in R was exported to Excel, with hashtags listed in one column and their frequency in another. The corresponding script in R has been provided in Multimedia Appendix 1 so that readers can replicate the analysis.

Because there was interest in detecting changes in the use of hashtags by schools of nursing after the results of *The Woodhull Study Revisited* were published in Fall 2018, the steps described above were repeated to split the 2016-2018 data set into two parts. The first covered September 29, 2016, through September 27, 2018 (the day that *The Woodhull Study Revisited* was published in the *Journal of Nursing Scholarship*), and the second covered September 28, 2018, through December 13, 2020. The same process outlined previously was used to analyze the data and generate frequency tables for the hashtags used during each time period of interest.

## Results

There were 6866 different hashtags used in the 2016-2020 data set. All hashtags that had been used 100 times or more across the entire corpus of tweets in the data set were identified, and these 71 hashtags were characterized as being those with the highest frequency of use by the schools of nursing in the study. These 71 hashtags were used a total of 26,243 times in the 2016-2020 data set, as detailed in Table 6. Among the 6866 different hashtags appearing in the 2016-2020 data set, 3774 were used only once and 6178 were used 10 or fewer times.

**Table 6:** Hashtags used 100 times or more in the 2016-2020 data set.

Hashtag	Number of times used
#nursing	3259
#pennnursing	1980
#healthcare	1265
#gwu	1192
#nurses	991
#covid19	895
#umson	887
#nurse	857
#jhson	606
#conhi	587
#nursingschool	565
#dnp	535
#vandygram	452
#nursesweek	451
#emorynursing	444
#bsn	442
#canenurse	419
#uabson	374
#npslead	372
#msn	358
#umichnursing	353
#tbt	348
#pennnursinginnovation	347
#volnurse	335
#simulation	287
#fpbnursing	279
#phd	260
#runursing	247
#gocougs	245
#raisehigh	232
#research	230
#icymi	226
#np	218
#cunursing	215
#vusn	215
#health	210
#hiv	205
#mentalhealth	204
#buckeyenurses	200
#nursepractitioner	199
#virginia	196
#yearofthenurse	185
#wegotthis	173
#veterans	170
#buckeyenurse	169
#nashville	164
#gohopnurse	161
#fau	160
#innovation	156
#amrchat	154
#uic	150
#npweek	149
#icowhi16	144
#jhuson	143
#givingtuesday	141
#meninnursing	136
#cwru	132
#huskynurses	132
#prerequisites	125
#globalhealth	122
#ahcj19	118
#bestgradschools	115
#nyu	115
#huskynurse	112
#opioid	111
#nursingstudent	109
#nurseleader	107
#nursingresearch	103
#nationalnursesweek	102
#umich	102
#uofunursing	101

When the data set was divided into two parts to detect changes in the use of hashtags by schools of nursing after the results of *The Woodhull Study Revisited* were published, the findings were similar to those of the analysis of the data set as a whole. There were 27 hashtags that had been used 100 times or more in the September 29, 2016, to September 27, 2018, data set. Among the 3307 different hashtags appearing in this data set, 1806 (54.6%) were used only once and 3028 (91.6%) were used 10 or fewer times. In comparison, there were 47 hashtags that had been used 100 times or more in the September 28, 2018, to December 13, 2020, data set. Among the 4812 different hashtags appearing in this data set, 2716 (56.4%) were used only once and 4350 (90.4%) were used 10 or fewer times. Tables 7 and 8 provide details on the hashtags used 100 times or more during each time period.

**Table 7:** Hashtags used 100 times or more before *The Woodhull Study Revisited*.

Top hashtags (September 29, 2016-September 27, 2018)	Number of times used
#nursing	1671
#pennnursing	1017
#gwu	671
#umson	530
#healthcare	516
#nurses	507
#nurse	409
#jhson	402
#conhi	393
#emorynursing	252
#nursingschool	243
#bsn	232
#nursesweek	227
#tbt	205
#buckeyenurses	177
#dnp	164
#volnurse	156
#amrchat	154
#icowhi16	144
#jhuson	143
#research	136
#buckeyenurse	133
#health	128
#canenurse	111
#wegotthis	109
#cunursing	106
#virginia	104

**Table 8:** Hashtags used 100 times or more after *The Woodhull Study Revisited*.

Top hashtags (September 28, 2018-December 13, 2020)	Number of times used
#nursing	1588
#pennnursing	963
#covid19	895
#healthcare	749
#gwu	521
#nurses	483
#vandygram	449
#nurse	448
#dnp	371
#umson	357
#umichnursing	353
#npslead	350
#nursingschool	322
#uabson	313
#canenurse	308
#msn	275
#pennnursinginnovation	264
#simulation	229
#raisehigh	228
#nursesweek	224
#bsn	210
#phd	205
#jhson	204
#conhi	194
#emorynursing	192
#yearofthenurse	185
#fpbnursing	182
#volnurse	179
#runursing	177
#vusn	171
#gocougs	163
#gohopnurse	161
#nashville	161
#mentalhealth	151
#tbt	143
#np	141
#fau	132
#icymi	129
#nursepractitioner	128
#meninnursing	127
#ahcj19	118
#hiv	115
#npweek	112
#cunursing	109
#cwru	104
#veterans	102
#uofunursing	101

### Typology of Frequently Used Hashtags

Using Excel, a thematic analysis was conducted of the hashtags that were used 100 times or more in the 2016-2020 data set. Collectively, the 71 hashtags were used a total of 26,243 times. To conduct the thematic analysis, the list of 71 frequently used hashtags was considered and similarities were assessed. As similarities were identified, the hashtags were grouped into categories, and this process of coding (and recoding) hashtags was continued until there were six categories that explained the vast majority of the hashtags. A seventh category was added to capture the assortment of hashtags that did not lend themselves to categorization. The following seven types of hashtags emerged during the process of thematic analysis: (1) Nursing, hashtags about nurses, nursing, nursing degrees, nursing licenses, etc; (2) Schools, hashtags about universities, schools, colleges, mascots, or locations; (3) Illness/disease/condition, hashtags about illnesses, diseases, conditions, or awareness day/month; (4) Population, hashtags about populations that nurses serve; (5) Health, hashtags about health care, health, global health, etc; (6) Conference or tweet chat, hashtags about conferences or specific Twitter chats for health care professionals; (7) Something else, hashtags that did not fit into one of the other six categories. Table 9 lists the hashtags contained in each of the seven categories.

**Table 9:** Hashtag typology of the 2016-2020 data set.

Category	Description of the category	Hashtags	Number of times used
Nursing	About nurses, nursing, nursing degrees, nursing licenses, etc	#bsn, #dnp, #meninnursing, #msn, #nationalnursesweek, #np, #npslead, #npweek, #nurse, #nurseleader, #nursepractitioner, #nurses, #nursesweek, #nursing, #nursingresearch, #nursingschool, #nursingstudent, #phd, #prerequisites, #simulation, and #yearofthenurse	9810
Schools	About universities, schools, colleges, mascots, or locations	#bestgradschools, #buckeyenurse, #buckeyenurses, #canenurse, #cunursing, #cwru, #emorynursing, #fau, #fpbnursing, #gocougs, #gohopnurse, #gwu, #huskynurse, #huskynurses, #jhson, #jhuson, #nashville, #nyu, #pennnursing, #pennnursinginnovation, #raisehigh, #runursing, #uabson, #uic, #umich, #umichnursing, #umson, #uofunursing, #vandygram, #virginia, #volnurse, and #vusn	10,974
Illness/disease/condition	About illnesses, diseases, conditions, or awareness day/month	#covid19, #hiv, and #opioid	1211
Population	About populations that nurses serve	#veterans	170
Health	About health care, health, global health, etc	#globalhealth, #health, #healthcare, and #mentalhealth	1801
Conference or tweet chat	About conferences or specific Twitter chats for health care professionals	#ahcj19, #amrchat, #conhi, and #icowhi16	1003
Something else	Hashtags that did not fit into one of the other six categories	#givingtuesday, #icymi, #innovation, #research, #tbt, and #wegotthis	1274

For the purposes of this study, the seven types of hashtags were considered to be either inward facing (“intercom hashtags”) or outward facing (“megaphone hashtags”). Intercom hashtags were those intended to invite attention from/interaction with nurses, members of the university/school community, or attendees at a nursing conference or Twitter chat. Megaphone hashtags were those intended to invite attention from/interaction with people such as journalists, policymakers, and the general public.

The intercom hashtag types were as follows: nursing (hashtags about nurses, nursing, nursing degrees, nursing licenses, etc); schools (hashtags about universities, schools, colleges, mascots, or locations); and conference or tweet chat (hashtags about conferences or specific Twitter chats for health care professionals). The megaphone hashtag types were as follows: illness/disease/condition (hashtags about illnesses, diseases, conditions, or awareness day/month); population (hashtags about populations that nurses serve); health (hashtags about health care, health, global health, etc); and something else (hashtags that did not fit into one of the other six categories).

The vast majority of the 71 hashtags that were used 100 times or more in the 2016-2020 data set can be categorized as intercom hashtags (inward-facing hashtags focused on in-group discussion within and about the profession). Collectively, nursing hashtags (n=9810, 37.4%), school hashtags (n=10,974, 41.8%), and conference or tweet chat hashtags (n=1003, 3.8%) comprised 83.0% (n=21,787) of the 26,243 times that the 71 frequently used hashtags occurred in the data set.

In contrast, few of the 71 hashtags that were used 100 times or more in the 2016-2020 data set can be categorized as megaphone hashtags. Collectively, health hashtags (n=1801, 6.9%), illness/disease/condition hashtags (n=1211, 4.6%), and population hashtags (n=170, 0.7%) comprised 12.1% (n=3182) of the 26,243 times that the 71 frequently used hashtags occurred in the data set. When the “something else” hashtags (5%) were added, the total of megaphone hashtags was approximately 18% of the 26,243 times that the 71 frequently used hashtags occurred in the data set.

When the data set was divided into two parts to detect changes in the use of hashtags by schools of nursing after the results of *The Woodhull Study Revisited* were published, the findings were similar to those of the analysis of the data set as a whole, with one notable exception. Prior to the publication of *The Woodhull Study Revisited* on September 27, 2018, none of the hashtags that were used 100 times or more pertained to an illness, disease, or condition. In the 2 years after the publication of *The Woodhull Study Revisited*, 7% of the frequently used hashtags pertained to an illness, disease, or condition. Further analysis revealed that this shift was attributable to the use of the following two hashtags: #covid19 (n=895) and #hiv (n=115).

### Missed Opportunities for Tweeting About Trending Topics

Of the 6866 different hashtags appearing in the 2016-2020 data set, 6178 were used 10 times or less. These seldom-used hashtags included a number of hashtags that were widely used on Twitter during the time period covered by this study. Table 10 contains a list of some of these hashtags along with the number of times each hashtag was used in the 2016-2020 data set.

**Table 10:** Missed opportunities to use hashtags of public interest.

Topic and hashtag		Number of times used in the 2016-2020 data set
**Racism, racial bias, and racial justice**		
	#racism	10
	#blacklivesmatter	9
	#antiracism	6
	#blm	6
	#bias	3
	#implicitbias	1
	#racialbias	1
	#unconsciousbias	1
	#systemicracism	1
	#racialjustice	1
**Sexism, sexual harassment, and rape**		
	#sexualharassment	2
	#rape	2
	#sexism	1
	#timesuphealthcare	1
**Politics**		
	#electionday	9
	#vote	8
	#election2020	2
	#election	2
	#trump	2
	#election2016	1
	#presidentialdebate2020	1
**LGBTQ^a^+ health**		
	#lgbtqhealth	2
	#homophobia	1
	#heterosexism	1
	#transhealth	1
**Cancer**		
	#lungcancer	8
	#pancreaticcancer	3
	#colorectalcancer	3
	#ovariancancer	2
	#skincancer	1
	#pediatriccancer	1
	#livercancer	1
	#childhoodcancer	1
**Other diseases and conditions**		
	#kidneydisease	6
	#hepatitis	2
	#arthritis	2
	#hearingloss	2
	#parkinsons	1
	#als	1
**Sexual health**		
	#sexualhealth	7
	#sexuality	1
	#abortion	1
	#condom	0
	#birthcontrol	0
	#familyplanning	0
**End of life**		
	#death	6
	#grief	2
	#advancedirective	1
	#livingwill	1
	#dying	0

^a^LGBTQ: lesbian, gay, bisexual, transgender, gender non-conforming, queer and/or questioning.

## Discussion

Although the top 44 schools of nursing have an active social media presence on Twitter, collectively, their use of hashtags functions more like an intercom to communicate with other nurses rather than a megaphone to invite attention from and dialogue with journalists, policy makers, and the general public. Because intercom hashtags are both inward facing and overused, they are of minimal use when it comes to drawing attention from and interacting with people outside of nursing. If schools of nursing want the media to showcase the voices of their faculty members as experts, schools of nursing need to be more strategic in their use of hashtags on Twitter. In order to accomplish this, schools of nursing need to increase their use of megaphone hashtags to connect the work of their faculty and students with topics and events of interest to the general public. For example, when topics like #guncontrol are trending, schools of nursing could tweet about the work their faculty members are doing in violence prevention.

On Twitter, schools of nursing have a unique opportunity to amplify the voices of their faculty members on health-related topics of widespread public interest like the impact of systemic racism on health, gun violence, and access to care, among others. If schools of nursing continue to use mostly intercom hashtags on Twitter, they will have squandered a powerful opportunity to share their expertise beyond the boundaries of the discipline.

## Appendix

Multimedia Appendix 1 R script for generating the data frame of the most frequently used hashtags in the data set.
