# Addendum to the Acknowledgments: Comparison of Intercom and Megaphone Hashtags Using Four Years of Tweets From the Top 44 Schools of Nursing: Thematic Analysis

**DOI:** 10.2196/29823

**Published:** 2021-04-29

**Authors:** Kimberly Acquaviva

**Affiliations:** 1 School of Nursing University of Virginia Charlottesville, VA United States

In “Comparison of Intercom and Megaphone Hashtags Using Four Years of Tweets From the Top 44 Schools of Nursing: Thematic Analysis” (JMIR Nursing 2021;4(2):e25114 doi: 10.2196/25114), the author noted one error.

In the originally published paper, the Acknowledgments section contained the following line about the source of funds for the 2016-2018 Twitter data:

The September 29, 2016, to February 22, 2018, Twitter data for this project were purchased with funds provided by the George Washington University School of Nursing’s Center for Health Policy and Media Engagement.

To increase clarity regarding the amount and original sources of funding provided for the purchase of data, this has been corrected to:

The September 29, 2016, to February 22, 2018, Twitter data for this project were purchased by the George Washington University School of Nursing’s Center for Health Policy and Media Engagement for $1000 with funds received from the Gordon and Betty Moore Foundation, Robert Wood Johnson Foundation, Beatrice Renfield Foundation, Sigma Theta Tau International, American Association of Critical-Care Nurses, Donald and Barbara Jonas Foundation, National League for Nursing, OnCourse Learning, American Association of Colleges of Nursing, American Organization of Nurse Executives, and Wolters Kluwer Health. No funding was provided for this study beyond the $1000 used for the purchase of data.

The correction will appear in the online version of the paper on the JMIR website on April 29, 2021, together with the publication of this correction notice. Because this was made after submission to PubMed, PubMed Central, and other full-text repositories, the corrected article has also been resubmitted to those repositories.

