# Supplementary material for: Comparison of Intercom and Megaphone Hashtags Using Four Years of Tweets From the Top 44 Schools of Nursing: Thematic Analysis
Source: JMIR Nurs. 2021 Apr 20;4(2):e25114. doi: 10.2196/25114 (PMC8279434; doi:10.2196/25114)
Supplement: Multimedia Appendix 1 [file nursing_v4i2e25114_app1.docx]

*## Save the Excel file called “tweets.xlsx” and then upload the Excel file into R*

*library(readxl)*

*tweets <- read_excel("Desktop/tweets.xlsx")*

*View(tweets)*

*## Use elements of Van Horn and Beveridge’s coding for cleaning the texts strings in the dataset. First, homogenize the character encoding to remove strings of nonsense characters indicating the presence of emojis in the source tweets. This will convert character encoding to Unicode UTF-8*

*tweets$Text <- iconv(tweets$Text, "", "UTF-8")*

*## Remove capitalization in tweets by turning everything into lowercase*

*tweets$Text <- tolower(tweets$Text)*

*## Remove URLs from the tweets*

*tweets$Text <- gsub("(http|https)([^/]+).*", " ", tweets$Text)*

*## Collapse extra whitespace into single space characters*

*tweets$Text <- gsub("[[:space:]]+", " ", tweets$Text)*

*## Identify the hashtags present in the dataset*

*hash.regexp <- "#[[:alpha:]][[:alnum:]_]+"*

*hashtags <- unlist(sapply(1:length(tweets$Text), function (x) { regmatches(tweets$Text[x],*

*gregexpr(hash.regexp, tweets$Text[x]))}))*

*## Count how many times each hashtag occurs*

*table(hashtags)*

*## Install the tidyverse package*

*install.packages("tidyverse")*

*## Attach the package*

*library(tidyverse)*

*## Store the table that you created with the `table` function to the variable my_table (don't do the sorting)*

*my_table <- table(hashtags)*

*## Convert the table into a data.frame*

*my_df <-*

*my_table %>%*

*as_tibble(.name_repair = "unique")*

*my_df %>%*

*write_csv(file = "~/Desktop/my_csv.csv")*
